# Invasive Pulmonary Aspergillosis in a Young Adult With Hyperimmunoglobulin E Syndrome and Hypogammaglobulinemia Following Rituximab Therapy

**DOI:** 10.7759/cureus.102562

**Published:** 2026-01-29

**Authors:** Gautam K Pandrangi, Ritika N Golechha, Logan R Mills, John T Brown, Nicholas Helmstetter

**Affiliations:** 1 Department of Medicine, Western Michigan University Homer Stryker M.D. School of Medicine, Kalamazoo, USA; 2 Department of Family Medicine, University of Michigan Health-West, Wyoming, USA; 3 Department of Pediatric and Adolescent Medicine, Western Michigan University Homer Stryker M.D. School of Medicine, Kalamazoo, USA

**Keywords:** adult immunodeficiency, cavitary lung disease, hyperimmunoglobulin e syndrome, hypogammaglobulinemia, intravenous immunoglobulins (ivig), invasive pulmonary aspergillosis, opportunistic fungal infection, rituximab therapy, signal transducer and activator of transcription 3 (stat3), structural lung disease

## Abstract

Hyperimmunoglobulin E syndrome (HIES) is a rare primary immunodeficiency marked by elevated IgE levels, recurrent skin and pulmonary infections, and immune dysregulation. While typically diagnosed in childhood, adult presentations can occur, often complicated by structural lung disease, opportunistic infections, and malignancies. We report a 37-year-old female with signal transducer and activator of transcription 3 (*STAT3*)-deficient HIES, diffuse large B-cell lymphoma (DLBCL) in remission, rituximab-induced hypogammaglobulinemia, and recurrent infections, who presented with acute-on-chronic dyspnea. Imaging revealed a large cavitary lesion in the left upper lobe. Despite 30 days of appropriate antibiotics targeting methicillin-resistant* Staphylococcus aureus* (MRSA) and gram-negative organisms, her respiratory status deteriorated, necessitating intubation. Subsequent bronchoalveolar lavage and serum galactomannan testing confirmed invasive pulmonary aspergillosis (IPA) as the etiology of her deterioration, prompting combination antifungal therapy. This case highlights the importance of maintaining a broad differential diagnosis in immunocompromised patients with worsening respiratory symptoms despite antibiotics. Early consideration and testing for fungal infections in high-risk populations can prevent diagnostic delay and improve outcomes.

## Introduction

Hyperimmunoglobulin E syndrome (HIES), formerly known as Job syndrome, is a heterogeneous group of primary immunodeficiencies characterized by markedly elevated IgE levels and recurrent infections [1]. The autosomal dominant form is most commonly caused by dominant-negative mutations in the signal transducer and activator of transcription 3 (*STAT3*) gene, resulting in multisystem disease involving the immune system, skeletal and integumentary system, dentition, and connective tissues [2]. *STAT3* mutations impair T helper 17 (Th17) cell differentiation, thereby compromising neutrophil recruitment. Keratinocytes and bronchial epithelial cells rely on Th17 cytokines, in combination with classical proinflammatory signals, to produce anti-staphylococcal factors, including chemokines and antimicrobial peptides [3]. This mechanism explains the selective vulnerability to skin and pulmonary infections observed in HIES [3].

Although HIES typically presents in childhood, recognition in adult patients is essential. Progressive immune dysfunction and cumulative pulmonary damage increase susceptibility to severe and opportunistic infections. Thus, this case aims to highlight the complex interplay of primary immunodeficiency, secondary immunosuppression, and structural lung disease resulting in life-threatening invasive pulmonary aspergillosis (IPA) in the setting of *STAT3*-deficient HIES.

## Case presentation

A 37-year-old female with autosomal dominant *STAT3*-deficient HIES, diffuse large B-cell lymphoma (DLBCL) in remission since 2024, chronic hypoxemic respiratory failure requiring 2 L/min of home supplemental oxygen, and rituximab-induced hypogammaglobulinemia presented with acute-on-chronic dyspnea. She had a documented allergy to trimethoprim-sulfamethoxazole (urticaria) and had self-discontinued intravenous immunoglobulin (IVIG) therapy several months prior to presentation.

Her medical history was significant for recurrent methicillin-resistant Staphylococcus aureus (MRSA) pulmonary infections resulting in progressive structural lung disease, including bronchiectasis and pneumatoceles. In March 2024, she was diagnosed with DLBCL. Her oncologic course was complicated by multiple episodes of necrotizing pneumonia and recurrent lung abscesses. Thus, she was only able to tolerate two out of six rounds of standard first-line treatment with rituximab, cyclophosphamide, doxorubicin, vincristine, and prednisone (R-CHOP) chemotherapy. Despite the abbreviated regimen, she achieved remission.

Following chemotherapy, IVIG was initiated to provide passive immunity and reduce infection burden. This was self-discontinued by the patient due to serum-sickness-like symptoms with diffuse myalgias and arthralgias. She was subsequently hospitalized for left upper lobe pneumonia, with cultures growing *Serratia* and *Klebsiella* *aerogenes*, and she was discharged on levofloxacin.

**Figure 1:**
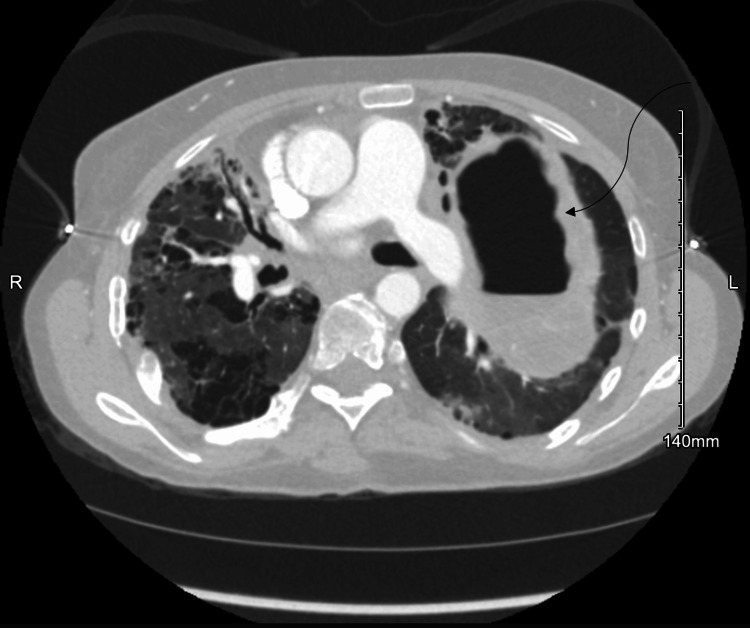
Computed Tomography Scan Demonstrating a Large Left Upper Lobe Cavitary Lesion With Surrounding Infiltrates The image shows a de-identified left upper lobe cavitary lesion in a patient with *STAT3*-deficient hyperimmunoglobulin E syndrome and secondary hypogammaglobulinemia. No identifying patient information is present, and informed consent for publication was obtained.

**Figure 2:**
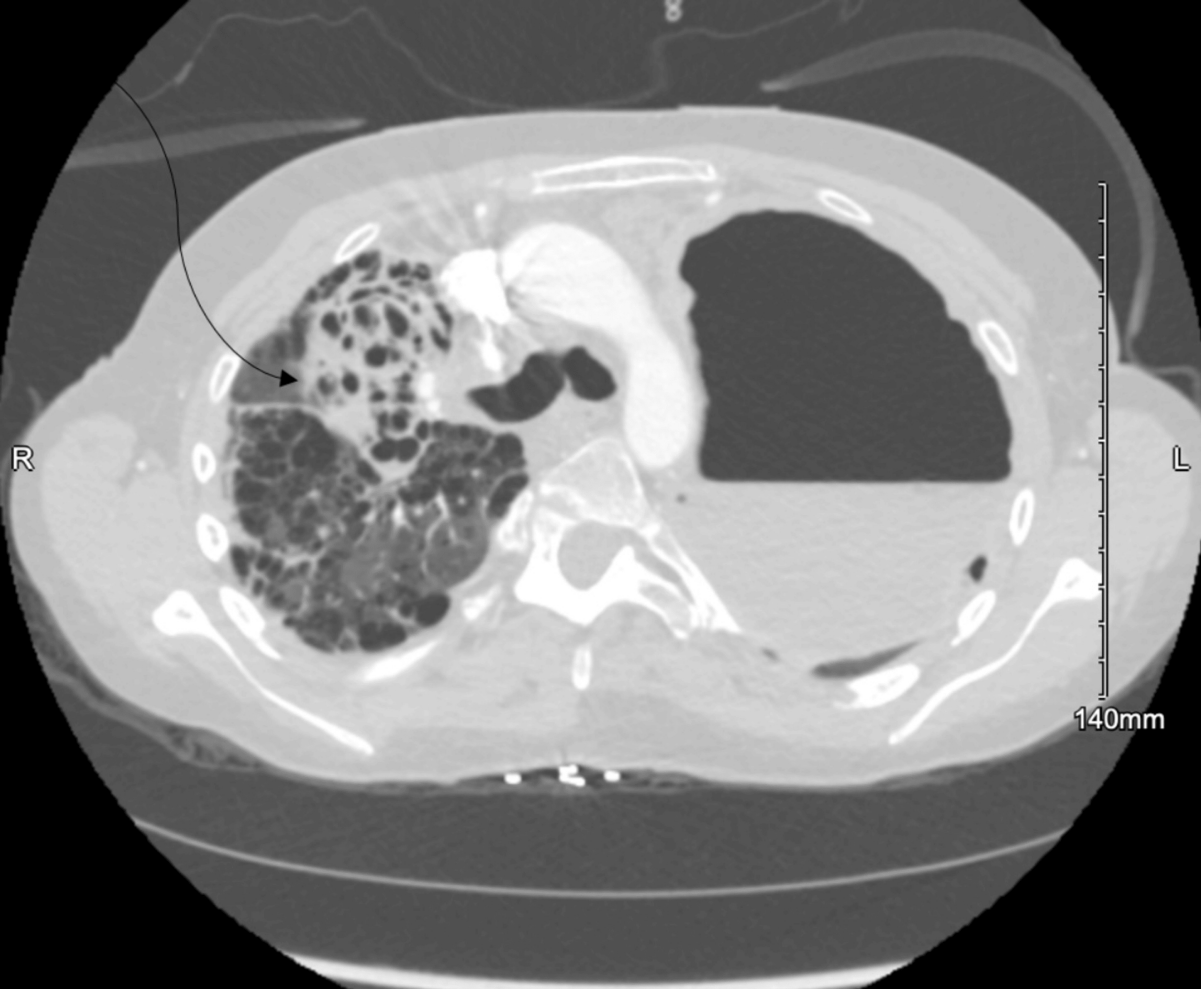
Computed Tomography Scan Demonstrating a Cavitary Lesion of the Right Upper Lobe and Apex The image shows a de-identified right upper lobe cavitary lesion in a patient with *STAT3*-deficient hyperimmunoglobulin E syndrome and secondary hypogammaglobulinemia. No identifying patient information is present, and informed consent for publication was obtained.

Despite adherence to treatment, her respiratory status worsened, with increasing oxygen requirements, prompting readmission. Computed tomography (CT) of the chest revealed a 12×12×9 cm cavitary lesion in the left upper lobe (Figure 1), consistent with a lung abscess. It also showed extensive areas of bronchiectasis, bronchial wall thickening, and airspace opacities in the right upper lobe and apex, suggesting a second cavitary lesion (Figure 2). Respiratory cultures grew MRSA and *Klebsiella pneumoniae*. Bronchial cultures also yielded *Candida albicans* and *Aspergillus *species, which were initially interpreted as colonizers.

**Figure 3:**
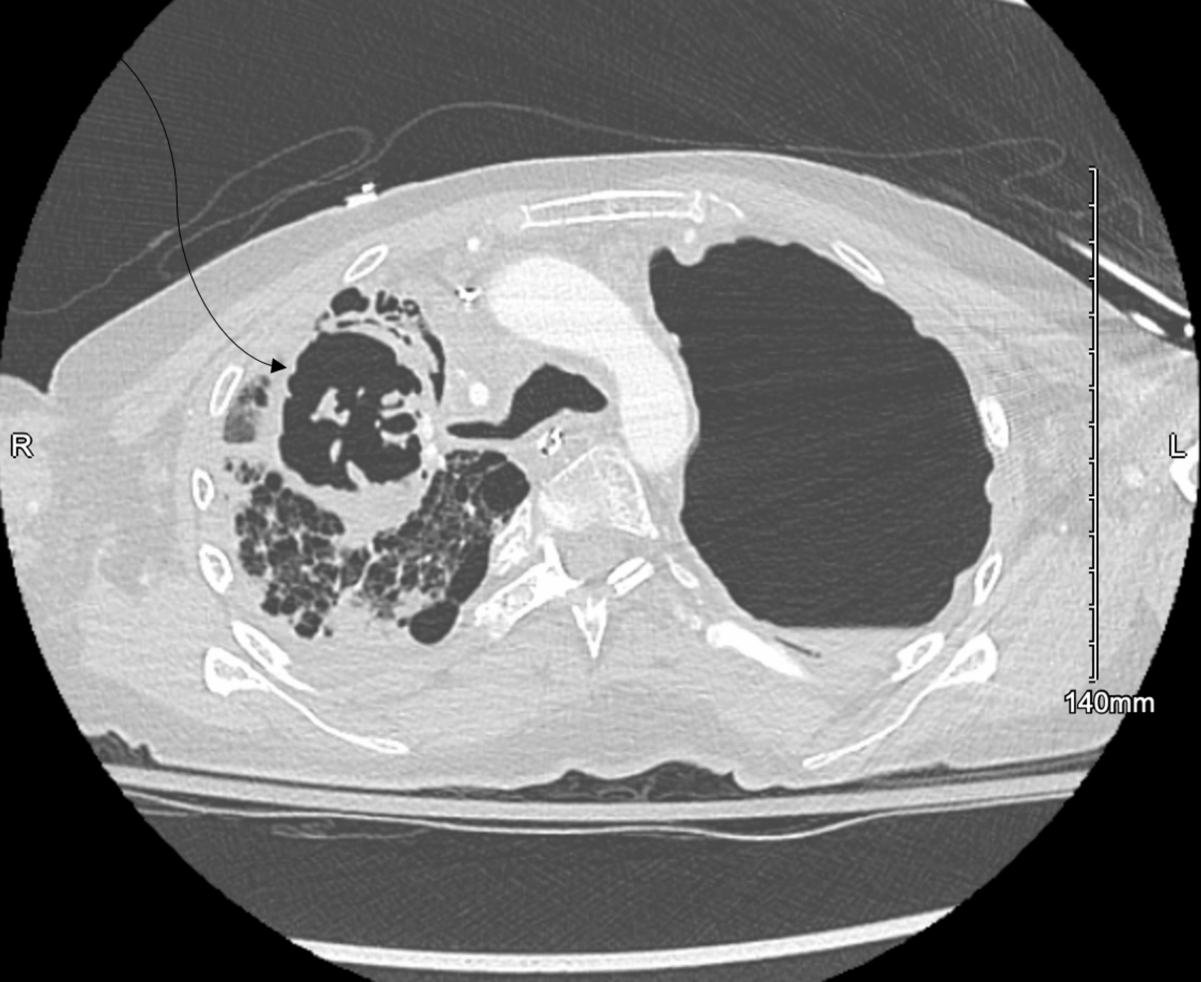
Follow-Up Computed Tomography Scan Demonstrating Further Progression of the Right Upper Lobe Cavitary Lesion The image shows a de-identified right upper lobe cavitary lesion in a patient with *STAT3*-deficient hyperimmunoglobulin E syndrome and secondary hypogammaglobulinemia. Compared with Figure 2, the cavitation shows increased complexity and wall thickening, suggestive of progression such as fungal infection or necrotizing pneumonia. No identifying patient information is present, and informed consent for publication was obtained.

Despite empiric therapy targeting MRSA and gram-negative organisms with vancomycin and cefepime, her respiratory failure progressed, necessitating intubation. Follow-up chest CT demonstrated increasing complexity of the right apical cavitation (Figure 3). Though initial bronchial cultures suggested *Aspergillus *species colonization, repeat tracheal aspirates grew MRSA in addition to *Aspergillus *species. Thus, in combination with the full clinical picture, bronchoalveolar lavage (BAL) and serum galactomannan testing were used to confirm IPA. Combination antifungal therapy with voriconazole and micafungin was initiated, along with continued antibacterial therapy with vancomycin, linezolid, and ertapenem. Her hospitalization lasted 51 days, including one month of antifungal treatment at standard adult dosing.

## Discussion

Baseline aspergillosis risk in *STAT3*-deficient HIES

*STAT3*-deficient HIES is associated with progressive pulmonary structural damage compared with other HIES variants [4]. Disease progression typically follows a three-step process: recurrent bacterial infections, development of structural lung abnormalities, and subsequent reinfections that further exacerbate pulmonary injury [5]. Early infections are predominantly *Staphylococcus aureus*, while later stages involve opportunistic pathogens such as *Aspergillus *species, *Mycobacterium tuberculosi*s, and *Pseudomonas aeruginosa* [5]. Our patient had reached the advanced disease stage, placing her at a higher risk for invasive fungal infections (Table 1).

**Table 1:** Baseline Aspergillosis Risk in Signal Transducer and Activator of Transcription 3-Deficient Hyperimmunoglobulin E Syndrome This table summarizes typical clinical features, disease progression, and pathogen involvement in patients with *STAT3*-deficient hyperimmunoglobulin E syndrome, highlighting risk factors for invasive pulmonary aspergillosis [2-5]. Abbreviations: *STAT3* = signal transducer and activator of transcription 3; Th17 = T helper 17

Feature	Description	Clinical Implication
*STAT3* mutation type	Autosomal dominant, dominant-negative	Impaired Th17 differentiation leading to compromised neutrophil recruitment
Typical disease progression	Recurrent bacterial infections → structural lung damage → recurrent infections	Cavitary lesions and bronchiectasis increase fungal infection risk
Early pathogens	Staphylococcus aureus	Predominantly bacterial infections in childhood
Late pathogens	*Aspergillus *species, *Mycobacterium tuberculosis*, and *Pseudomonas aeruginosa*	Opportunistic infections in advanced disease
Clinical relevance	Advanced structural lung disease	High risk for invasive pulmonary aspergillosis in adults

DLBCL and rituximab-induced hypogammaglobulinemia

Patients with *STAT3*-deficient HIES are predisposed to lymphomas due to underlying immune dysregulation, with DLBCL reported among the most common malignancies [6,7]. Our patient developed DLBCL and was treated with two cycles of R-CHOP (Table 2). Rituximab depletes CD20-positive B cells, which can result in prolonged hypogammaglobulinemia that may persist for up to 24 months, with sustained IgG reductions of four months or longer conferring a significantly increased risk of serious infections [8]. Notably, our patient presented nearly one year after cessation of R-CHOP therapy, illustrating the prolonged window of immunologic vulnerability that can persist despite oncologic remission.

**Table 2:** Diffuse Large B-Cell Lymphoma and Rituximab-Induced Hypogammaglobulinemia in Hyperimmunoglobulin E Syndrome This table summarizes reported malignancy risk, effects of rituximab-based therapy, and duration of immune suppression in patients with hyperimmunoglobulin E syndrome, with implications for immunoglobulin replacement therapy [6-8]. Abbreviations: HIES = hyperimmunoglobulin E syndrome; DLBCL = diffuse large B-cell lymphoma; IVIG = intravenous immunoglobulin; *STAT3* = signal transducer and activator of transcription 3

Feature	Description	Clinical Implication
Malignancy	Increased risk of lymphomas, especially DLBCL	Immune dysregulation predisposes to lymphoma
Therapy	Rituximab-based chemotherapy	CD20-positive B-cell depletion leads to prolonged hypogammaglobulinemia
Duration of immune suppression	Up to 24 months following rituximab	Increased susceptibility to serious infections
IVIG use	Not routinely indicated in *STAT3*-deficient HIES	Considered in secondary hypogammaglobulinemia with structural lung disease
Clinical challenge	Self-discontinuation due to myalgias	Loss of humoral protection; need alternative formulations or premedication

Although IVIG therapy is not routinely indicated in *STAT3*-deficient HIES, given that most patients retain baseline antibody production, this case represents a unique clinical context [9,10]. Recent chemotherapy, rituximab-induced hypogammaglobulinemia, structural lung disease, and recurrent infections created a compounded risk state that justified immunoglobulin replacement. Thus, IVIG was initiated to provide passive immunity and reduce infectious burden while maintaining IgG levels within the normal range. However, the patient self-discontinued IVIG due to myalgias and arthralgias, resulting in loss of critical humoral immune support and further increasing susceptibility to encapsulated bacteria and opportunistic pathogens [9]. Alternative strategies such as subcutaneous immunoglobulin, premedication protocols, or different IVIG formulations may have allowed continued immunoglobulin replacement while minimizing adverse effects [11,12].

Polymicrobial nature and diagnostic complexity

The polymicrobial nature of this patient’s infection reflects severely compromised host defense and structural lung disease, characteristic of advanced HIES, contributing to diagnostic complexity (Table 3). Initial respiratory cultures grew *Klebsiella*, MRSA, *Candida albicans*, and *Aspergillus *species. Bacterial pathogens were initially presumed causal, while* Candida* and *Aspergillus* were considered colonizers. Respiratory status continued to decline despite appropriate antibiotic therapy. Progression from bacterial pneumonia to invasive aspergillosis illustrates how bacterial infections can create favorable conditions for fungal superinfection in cavitary lesions [13]. This underscores the importance of continuously re-assessing treatment response and considering alternative superinfections in patients with worsening clinical status.

**Table 3:** Polymicrobial Infections and Diagnostic Complexity in Advanced Hyperimmunoglobulin E Syndrome This table summarizes the polymicrobial nature of respiratory infections in advanced hyperimmunoglobulin E syndrome and highlights diagnostic challenges associated with structural lung disease, including delayed recognition of invasive fungal infection and the need for repeated reassessment and multimodal diagnostics [13]. Abbreviations: BAL = bronchoalveolar lavage; MRSA = methicillin-resistant *Staphylococcus aureus*

Feature	Description	Clinical Implication
Polymicrobial cultures	*Klebsiella*, MRSA, *Candida albicans*, *Aspergillus *species	Diagnostic uncertainty in advanced structural lung disease
Initial interpretation	Bacterial pathogens treated; fungal organisms presumed colonizers	Delay in antifungal therapy
Clinical course	Progressive respiratory decline despite antibiotics	Suggests a superimposed invasive fungal infection
Diagnostic complexity	Overlapping colonization and infection	Requires reassessment and multimodal diagnostics

Invasive aspergillosis carries mortality rates exceeding 50% in critically ill patients if not adequately treated, making early diagnosis critical [14]. Complex cases may require multiple diagnostic modalities, including cultures from multiple sites, imaging, BAL, and serum markers such as galactomannan, beta-D-glucan, and PCR assays [15]. The American Thoracic Society supports serum and BAL galactomannan testing as alternatives when tissue biopsy is not feasible [15]. Lung biopsy should be pursued if noninvasive testing fails and is clinically appropriate.

Management challenges and treatment considerations

The Infectious Diseases Society of America (IDSA) and American Thoracic Society recommend voriconazole as first-line therapy for invasive aspergillosis, with isavuconazole and liposomal amphotericin B as alternatives [16,17]. Given the high mortality in critically ill patients, combination therapy with voriconazole and an echinocandin may be considered in high-risk cases [17]. This patient required intubation due to acute hypoxic respiratory failure, underscoring disease severity. She received micafungin and voriconazole for one month, alongside cefepime, vancomycin, linezolid, and ertapenem for bacterial coverage.

Prophylaxis gaps and preventive strategies

This case highlights critical gaps in infection prevention. The National Comprehensive Cancer Network (NCCN) recommends mold-active antifungal prophylaxis for patients at intermediate to high infection risk, including those undergoing or recently completing intensive chemotherapy [18]. Given her HIES, structural lung disease, and planned R-CHOP therapy, this patient would have been an ideal candidate for primary antifungal prophylaxis [5]. The IDSA recommends mold-active triazoles when the risk of invasive aspergillosis exceeds 6%, a threshold likely met by this patient [16]. Guidelines may benefit from clarification that *STAT3*-deficient HIES patients with structural lung damage should receive extended antimicrobial coverage, including antifungals [16].

Discontinuation of IVIG due to myalgias represents a missed opportunity for ongoing humoral immune support. Alternative IVIG preparations, subcutaneous immunoglobulin, or premedication strategies could have maintained prophylaxis while mitigating adverse effects [19]. Secondary prophylaxis following initial bacterial infections during chemotherapy should also be considered. Given a sulfa-drug allergy limiting *Pneumocystis jirovecii *prophylaxis, alternatives such as atovaquone or dapsone could have been employed [20].

## Conclusions

This case highlights the potentially severe consequences when multiple layers of immune dysfunction overlap: primary immunodeficiency (*STAT3*-deficient HIES), structural lung disease, secondary immunosuppression (rituximab-induced hypogammaglobulinemia), and gaps in prophylaxis. Optimal outcomes depend on rapid recognition, prompt antifungal therapy, and surgical intervention when needed. Long-term care requires vigilance for recurrent infections, proactive pulmonary management, and strategic use of antifungal prophylaxis during periods of heightened immunologic risk.

Several important diagnostic and management principles emerge from this presentation. In immunocompromised patients with progressive respiratory symptoms despite adequate antibacterial treatment, clinicians should maintain a broad differential diagnosis that includes invasive fungal disease. Early diagnostic evaluation with fungal biomarkers, advanced imaging, and tissue sampling, when feasible, may facilitate earlier diagnosis and improve outcomes. Optimal management is best achieved through a multidisciplinary approach involving immunology, infectious diseases, oncology, and pulmonary medicine, allowing coordinated decisions regarding antimicrobial therapy, immunoglobulin replacement strategies, and procedural interventions. Prophylactic measures should be strongly considered in patients with overlapping risk factors for invasive fungal infections. When standard therapies are contraindicated or poorly tolerated, individualized strategies, including alternative immunoglobulin formulations or modified premedication protocols, may be necessary to ensure continuity of care. Recognition of this compounded high-risk state is critical to minimizing diagnostic delays and reducing morbidity and mortality.
